# Kyste hydatique du pancréas révélé par une pancréatite aigüe: à propos d'un cas

**DOI:** 10.11604/pamj.2015.22.166.6242

**Published:** 2015-10-21

**Authors:** Hedfi Mohamed, Sridi Azza, Abdelhedi Chrif, Sassi Karim, Chouchen Adnen

**Affiliations:** 1 Service de Chirurgie Générale, Hôpital des FSI, Marsa, Tunisie

**Keywords:** Pancréas, kyste hydatique, diagnostique, chirurgie, Pancreas, hydatid cyst, diagnostic, surgery

## Abstract

La localisation pancréatique du kyste hydatique est exceptionnelle, même dans les pays où la maladie hydatique sévit à l’état endémique. Nous rapportons une observation de kyste hydatique du pancréas révélée par une pancréatite aigue. Le diagnostic était porté sur les données du scanner abdominal qui objectivait une masse kystique de la queue du pancréas adhérente au hile splénique. Le traitement chirurgical avait consisté en une splénopancréatectomie gauche. A travers cette observation et une revue de la littérature, nous discutons les difficultés diagnostiques et thérapeutiques de cette localisation rare du kyste hydatique.

## Introduction

La localisation pancréatique du kyste hydatique est très rare même dans les pays endémiques. La symptomatologie clinique est souvent insidieuse. Elle est dominée par les douleurs abdominales, le syndrome de masse et l'ictère, d'intensité variable en fonction du siège du kyste. Certaines complications évolutives bien que rares peuvent être révélatrices. Il peut s'agir d'une perforation, d'une hémorragie ou d'une fistulisation à l'origine de syndrome infectieux ou de douleurs abdominales récurrentes. Lorsqu'il est isolé, le kyste hydatique du pancréas peut poser un problème diagnostique avec les autres tumeurs kystiques du pancréas, malgré l'apport des moyens modernes d'imagerie. Les auteurs rapportent une observation rare de kyste hydatique du pancréas révélé par une pancréatite aiguë. Ils étudient les particularités cliniques, para cliniques et thérapeutiques de cette affection rare en soulignant les difficultés diagnostiques qu'elles posent.

## Patient et observation

**Figure 1:**
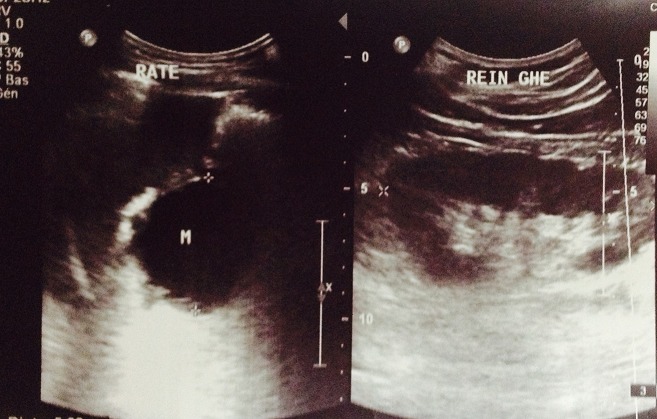
Pancréas augmenté de taille avec une formation kystique de la queue de 54 mm

**Figure 2:**
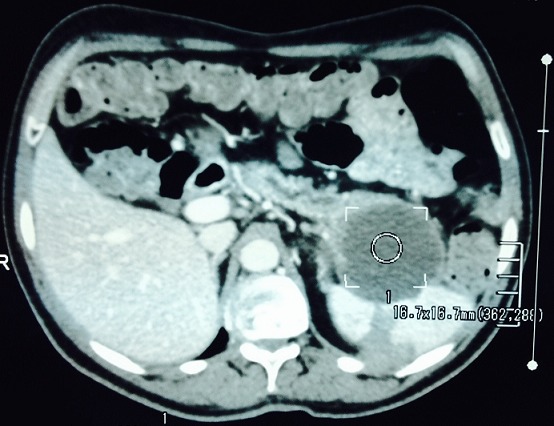
TDM coupe transversale: formation kystique de 6 cm uniloculaire de la queue du pancréas adhérant intimement à la rate avec dilatation modérée du canal de Wirsung

**Figure 3:**
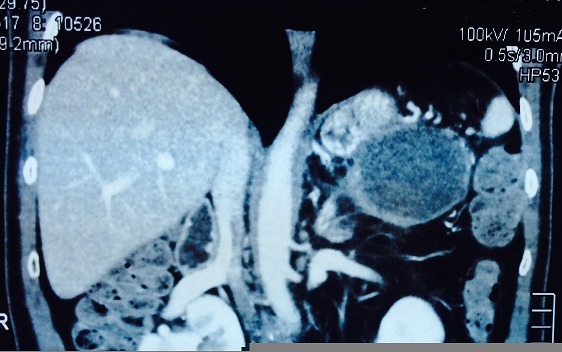
TDM coupe longitudinale montrant la lésion kystique uniloculaire

Mr M.D âgé de 51 ans sans antécédents pathologiques hospitalisé pour douleurs épigastrique associées à des vomissements évoluant depuis 6 mois qui s’étaient accentuées la veille de son admission. L'examen avait trouvé un patient avec un état général conservé subfébrile à 37,9° présentant une sensibilité épigastrique et de l'hypochondre droit à la palpation de l'abdomen, mais sans masse palpable. Il n'y avait pas d'ictère. La biologie avait noté, une amylasémie a 10 fois la normale et une hyperleucocytose à 21000el/mm^3^. Les bilans hépatique et rénal étaient sans anomalies. Une échographie abdominale réalisée en urgence avait mis en évidence un pancréas augmenté de taille avec une formation kystique de la queue du pancréas de 54 mm. La vésicule était alithiasique. Il n'y avait pas de dilatation des voies biliaires (Figure 1). La tomodensitométrie avait objectivé un pancréas atrophique, siège au niveau de sa portion caudale d'une formation kystique de 6 cm uniloculaire adhérant intimement à la rate. Il existait une dilatation modérée du canal de Wirsung mais sans dilatation de la voie biliaire principale (Figure 2, Figure 3).

**Figure 4:**
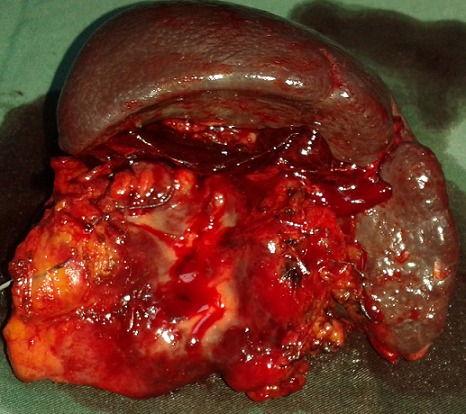
Pièce de splénopancréatectomie gauche

Le diagnostic retenu était celui d'une tumeur kystique du pancréas compliquée d'une pancréatite aiguë stade A de Balthazar. L’évolution clinique était favorable sous traitement médical antalgique et antiémétique. La sérologie hydatique et les marqueurs tumoraux (ACE, CA 19-9) étaient négatifs. Le diagnostic de tumeur kystique de la queue du pancréas était posé sans présomption de sa nature. Le malade a été opéré par voie médiane. L'exploration avait trouvé une masse kystique de la queue du pancréas, de nature hydatique adhérent intimement au pédicule splénique. Il a été réalisé une splénopancréatectomie gauche avec suture de la tranche de section pancréatique (Figure 4). Les suites opératoires étaient simples. L'examen anatomopathologique avait conclu à un kyste hydatique du pancréas.

## Discussion

Le kyste hydatique, échinococcose hydatique ou hydatidose, est une maladie parasitaire due aux œufs d´un ténia, l´*Echinococcus granulosus*. Elle s'intègre au sein des cestodes larvaires. C'est une zoonose complexe touchant de nombreuses espèces d'animaux. Elle affecte accidentellement l'homme qui s'insère comme hôte intermédiaire dans le cycle de l'helminthiase [1]. La localisation pancréatique du kyste hydatique est très rare [2, 3]. Elle représente 0,2% de l'ensemble des localisations abdominales de la maladie hydatique [4]. L'infestation du pancréas se fait par voie artérielle après passage des filtres hépatique et pulmonaire. La localisation pancréatique est isolée dans 91% des cas [2–5]. Le siège est céphalique dans 57% des cas, corporéal dans 24% des cas et caudal dans 19% des cas [4, 6]. Le kyste a un développement intra parenchymateux dans 35% des cas et périphérique dans 65% des cas [4, 7]. La taille peut varier de quelques millimètres à plusieurs centimètres [4, 5]. La symptomatologie clinique est variable et non univoque. Elle dépend de la taille et du siège du kyste[3, 6, 7] Il peut s'agir de douleurs abdominales (60% des cas) de l’étage sus-ombilicale, d'un ictère rétentionnel dans les formes céphaliques (34%) ou d'une masse abdominale palpable.

Des complications évolutives peuvent être révélatrices. Elles sont à type de suppuration, de rupture intra ou rétro-péritonéale, d'hémorragie ou encore une compression du pédicule splénique ou du tronc veineux splénomésaraique [5, 8]. L'hypertension portale est rencontrée dans 14% des cas, elle est souvent asymptomatique [4, 5, 9, 10]. La masse peut comprimer la voie biliaire principale dans les formes céphaliques. L'ouverture du kyste dans le tube digestif ou dans les voies biliaires est possible mais exceptionnelle (0,5% des cas) [2, 8–11]. La fistulisation dans le Wirsung peut être responsable de poussées de pancréatite aiguë récidivantes, de pancréatite chronique obstructive voire de wirsungorragie [5, 9, 12–14]. L’échographie, la tomodensitométrie et l'imagerie par résonnance magnétique permettent souvent de reconnaitre la lésion kystique pancréatique. Cependant la difficulté est de rattacher cette lésion à la maladie hydatique. La présence d'une autre localisation hydatique abdominale ou pulmonaire, le décollement de membrane kystique à l’échographie et la présence de calcifications arciformes sur les clichés d'abdomen sans préparations ou sur le scanner sont en faveur de l'origine hydatique [1, 12].

Les arguments épidémiologiques ainsi que la sérologie hydatique lorsqu'elles sont positives peuvent aider au diagnostic. Cependant, la négativité de cette dernière n’élimine pas la nature hydatique d'une masse kystique pancréatique. Ainsi, le diagnostic différentiel se posera essentiellement avec le cystadénome séreux (CS) et le cystadénome mucineux (CM). Classiquement l'aspect radiologique de ces tumeurs est caractérisé par la présence, au sein de la masse kystique, de cloisons qui se rehaussent après injection de produit de contraste [4, 5, 15]. Cependant, cet aspect n'est pas constant et l'on peut retrouver des calcifications arciformes dans les CM et des calcifications centrales dans les CS avec des cloisons dont la prise de contraste n'est pas toujours très nette [4, 5, 12, 15]. En cas de persistance d'un doute diagnostique, le recours à l’écho-endoscopie est d'un grand apport du fait d'une meilleure étude du contenu de la tumeur kystique surtout pour les localisations céphaliques [6, 8, 10, 13, 15]. La place des investigations invasives comme le cathétérisme rétrograde du canal de Wirsung ou les opacifications vasculaires est réduite aux formes compliquées [6, 14]. La cytoponction diagnostique est formellement contre indiquée à cause du risque de dissémination rétro- ou intra péritonéale. Le traitement du kyste hydatique du pancréas est chirurgical. Le choix dépend du siège du kyste et de l'existence ou pas d'une fistule kysto-canalaire [14].

Dans tous les cas, si la nature hydatique d'une masse kystique du pancréas est suspectée, une protection du champ opératoire et de la paroi abdominale par une solution scolicide est obligatoire [4, 7, 15]. En l'absence de fistule, le traitement doit être simple avec une résection du dôme saillant, un drainage externe avec ou sans épiploplastie. [3, 6, 9, 11–15] En cas de fistule canalaire, le geste est fonction du siège de la lésion: si le kyste est corporéal et/ou caudal, une résection radicale a type de pancréatectomie gauche avec ou sans splénectomie est indiquée [4, 14]; si le kyste est céphalique, une anastomose kysto jéjunale sur une anse en Y ou une suture canalaire sur un drain tuteur trans-duodéno-papillaire peuvent être réalisées. Le choix dépendra de la taille de la fistule [14]. La duodénopancréatectomie céphalique est un geste disproportionné devant cette affection parasitaire bénigne [3, 4, 6, 14].

## Conclusion

L'hydatidose pancréatique est exceptionnelle. Elle pose souvent des difficultés diagnostiques devant une lésion kystique isolée, malgré la contribution et l'apport des moyens modernes d'imagerie médicale. Le traitement de cette affection est chirurgical. La technique de référence reste la résection du dôme saillant.
